# The Cytoskeletal Protein Zyxin Inhibits Retinoic Acid Signaling by Destabilizing the Maternal mRNA of the RXRγ Nuclear Receptor

**DOI:** 10.3390/ijms23105627

**Published:** 2022-05-17

**Authors:** Elena A. Parshina, Eugeny E. Orlov, Andrey G. Zaraisky, Natalia Y. Martynova

**Affiliations:** 1 Shemyakin-Ovchinnikov Institute of Bioorganic Chemistry of the Russian Academy of Sciences, 117997 Moscow, Russia; orlov_evgeny@yahoo.com (E.E.O.); azaraisky@yahoo.com (A.G.Z.); 2 Pirogov Russian National Research Medical University, 117997 Moscow, Russia

**Keywords:** zyxin, ybx1, mRNA, nuclear receptor, retinoic acid, *Xenopus*, development

## Abstract

Zyxin is an LIM-domain-containing protein that regulates the assembly of F-actin filaments in cell contacts. Additionally, as a result of mechanical stress, Zyxin can enter nuclei and regulate gene expression. Previously, we found that Zyxin could affect mRNA stability of the maternally derived stemness factors of Pou5f3 family in *Xenopus* *laevis* embryos through binding to Y-box factor1. In the present work, we demonstrate that Zyxin can also affect mRNA stability of the maternally derived retinoid receptor Rxrγ through the same mechanism. Moreover, we confirmed the functional link between Zyxin and Rxrγ-dependent gene expression. As a result, Zyxin appears to play an essential role in the regulation of the retinoic acid signal pathway during early embryonic development. Besides, our research indicates that the mechanism based on the mRNA destabilization by Zyxin may take part in the control of the expression of a fairly wide range of maternal genes.

## 1. Introduction

The Lim-domain protein Zyxin is mainly known for its function as a scaffolding protein involved into the assembly of the actin filaments in cell contacts [1,2,3,4]. Additionally, Zyxin can play a role of a mechanotransducer, which leaves cell contacts in case of mechanical stresses and enters the nucleus where it operates as a transcriptional regulator [5,6]. Recent works have also confirmed that such roles are played by Zyxin during embryogenesis [7,8,9]. All this make Zyxin as a promising candidate for the role of one of the proteins that couple the embryonic morphogenetic movements with gene expression. 

Using RNA-seq, we previously analyzed the effects of *Zyxin* knock-down on the transcriptomes of the dorsal tissue cells of the *Xenopus laevis* neurula stage embryos [10]. We found out that *Zyxin* downregulation increases expression of the *pou5f3* family genes (paralogs of Pou5f1/Oct4 in mammals), which are responsible for maintenance of stem cell status [10]. As we further demonstrated, this effect is based on stabilization of the maternal transcripts of *pou5f3* genes by Y-box RNA-binding protein Ybx1. In the cytoplasm, Zyxin binds Ybx1 resulting in dissociation of the latter from *pou5f3* mRNA, which increases its degradation. Thus, Zyxin in this context operates as a molecular switch between the undifferentiated totipotent state of the zygote and cell differentiation in the early embryo. 

To verify whether the revealed mechanism of the mRNA destabilization by Zyxin could also operates in case of transcripts of other maternal genes, we investigated in the present work a possible role of Zyxin in the regulation of mRNA stability of the Rxrγ retinoid receptor. Along with *pou5f3*, *Rxrγ* was identified in our previous screening as a gene whose mRNA level was strongly increased in embryos with the downregulated *Zyxin.* Noteworthy, *Rxrγ* mRNA, such as *pou5f3* transcripts, is accumulated maternally, but rapidly degrades after the mid-blastula transition (MBT) [11].

Retinoid X receptors (Rxrs) and retinoic acid receptors (Rars) belong to the class of Type II nuclear receptors [12]. They usually act as heterodimers and upon ligand binding occupy DNA as retinoic acid responsive elements (RARE) to drive retinoid responsive transcription [13]. Rxr-Rar heterodimers are activated by a group of naturally occurring retinoids, which bind Rar and/or Rxr with different affinities [14]. For instance, Rar binds 9-cis retinoic acid (9cRA) as well as all-trans retinoic acid (atRA) while Rxr binds only 9-cis retinoic acid (9cRA) [15]. Rxr alone can, however, form homodimers and drive transcription from Rxr-responsive elements (RXRE) upon binding with 9cRA [16]. In developmental processes, retinoic acid (RA) and its stereoisomers play an essential role as biologically active derivatives of retinol (vitamin A). Excess or deficiency of RA signaling during development leads to a variety of defects in morphogenesis, organogenesis, growth, apoptosis, or tissue homeostasis [17,18,19].

Six retinoid receptors are known in *Xenopus laevis*: xRars (Rarα, Rarβ, Rarγ), and xRxrs (Rxrα, Rxrβ, Rxrγ). At least four of them (xRxrα, xRxrγ, xRarα, and xRarγ) are expressed in the *X. laevis* egg. With exception of *xrarγ*, these maternal mRNAs are degraded before gastrulation. These four receptors provide a molecular basis for the pleiotropic effects of RA in early development. The differential response of these receptors to retinoid ligands and the expression patterns are consistent with their specific roles in early development [20,21]. 

As we demonstrate in the present work, Zyxin is indeed involved in the regulation of *rxr*γ mRNA stability in the *Xenopus laevis* embryo by the mechanism that we described previously for *pou5f3* mRNAs [10]. Namely, Zyxin destabilizes *rxrγ* mRNA via sequestering Ybx1 that otherwise binds and stabilizes this mRNA. Thus, besides revealing a previously unknown role of Zyxin in the regulation of the *rxrγ* mRNA level, our data indicate that the previously identified mechanism of mRNA destabilization via the interaction of Zyxin with Ybx1 could play the role of a more general mechanism regulating mRNA levels of maternal transcripts in early embryogenesis. 

## 2. Results

### 2.1. Downregulation of Zyxin Results in the Increase in rxrγ mRNA Level

First of all, to verify the data of our previous RNA-seq screening by another method, we compared *rxrγ* mRNA levels in the middle gastrula stage *Xenopus laevis* embryos with knocked down *Zyxin* and the control levels by qRT-PCR. To knock-down *Zyxin,* the embryos were injected with conventional antisense morpholino oligonucleotide (MO) to *Zyxin* mRNA. The control embryos were injected with *control* MO (see Supplementary Table S1).

**Figure 1:**
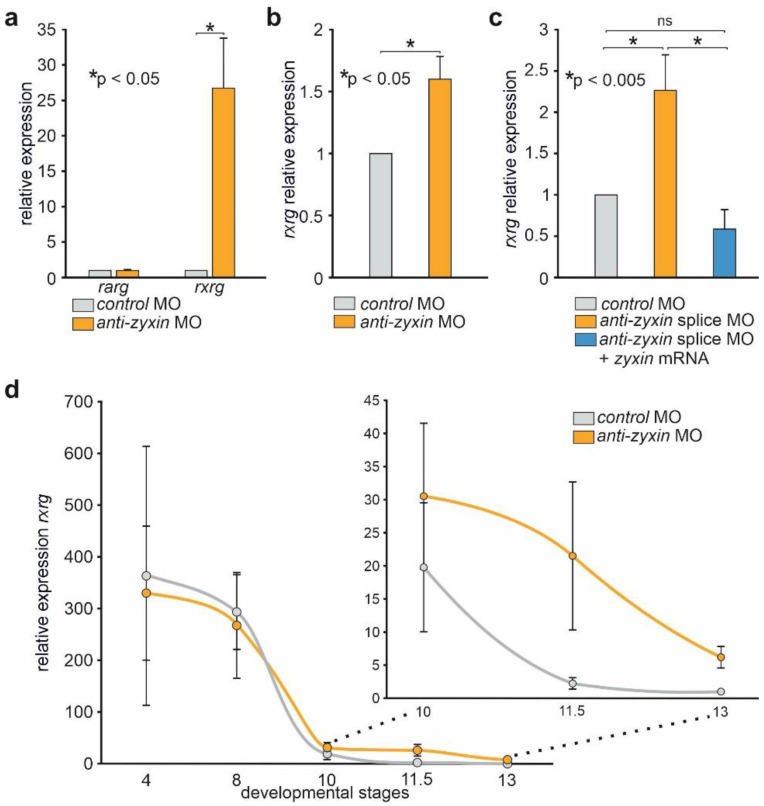
Downregulation of *Zyxin* results in an increase in the *rxrγ* mRNA level in the *Xenopus laevis* embryos. (**a**) Analysis by qRT-PCR of *rxrγ* and *rarγ* expression in the middle-gastrula stage *Xenopus laevis* embryos injected with the control and *anti-zyxin* MO. Here and below the transcript levels of the housekeeping genes *odc1* and *eef1a1* were used for the data normalization. Error bars indicate standard deviation. (**b**) Analysis by qRT-PCR of *rxrγ* expression at bud stage of *Danio rerio* embryos injected with the control and anti-*zyxin* splice MO. (**c**) Analysis by qRT-PCR of the rescue effect on *rxrγ* expression of *zyxin* mRNA. (**d**) Comparative analysis of the *rxrγ* transcripts levels in *Zyxin* knocked-down and control embryos at different stages of development. The inset shows at larger scale the data for stages from 10 to 13. The data presented are averaged from at least three independent experiments.

As a result, more than 20-fold higher *rxrγ* mRNA levels were detected in embryos injected with any of the aforementioned *anti-zyxin* MO compared to the control levels (Figure 1a). In contrast, the expression of zygotic gene *retinoid acid receptor gamma* (*rarγ)*, which was selected as an additional control, was not affected in this experiment. Importantly, a similar increase in *rxrγ* mRNA upon *Zyxin* down-regulation by MO was observed in the *Danio rerio* fish embryos, suggesting the evolutionary conservation of the effect (Figure 1b). The specificity of the MO activity on the *Xenopus laevis rxrγ* mRNA was confirmed in rescue experiments, in which we observed preservation of the basal level of the *rxrγ* mRNA concentration in embryos co-injected with *anti-zyxin* splice MO, which prevented maturation of this mRNA due to the binding to the exon3–intron3 boundary, and the synthetic *zyxin* mRNA (Figure 1c). In addition, we confirmed the efficiency of zyxin knock-down by MO, as well as the efficiency of its rescue by zyxin mRNA co-injection, analyzing the levels of Zyxin protein in these embryos (Figure S1).

To investigate changes of the *rxrγ* expression in embryos with downregulated *Zyxin* in more detail, we compared *rxrγ* expression in the control and *anti*-*zyxin* MO injected embryos at different developmental stages. At the stages prior to MBT, the mRNA levels of *rxrγ* were statistically indistinguishable in the control and *anti-zyxin* MO embryos (Figure 1d and Supplementary Figure S2). From MBT to early gastrula stage (stage 10), the mRNA levels also decrease similarly both in the controls and in *anti-zyxin* MO injected embryos (Figure 1d and Supplementary Figure S2). However, from gastrula stage 10 to stage 13 (early neurula), the decay of *rxrγ* mRNA in *anti*-*zyxin* MO embryos becomes significantly slower, eliciting significantly higher levels of *rxrγ* mRNA in the *anti-zyxin* MO injected embryos (Figure 1d and Supplementary Figure S2).

Thus, the data obtained suggest that Zyxin definitely plays a role in the process of the degradation of the maternally accumulated *rxrγ* mRNA.

### 2.2. Zyxin Prevents Binding of Ybx1 to rxrγ mRNA

**Figure 2:**
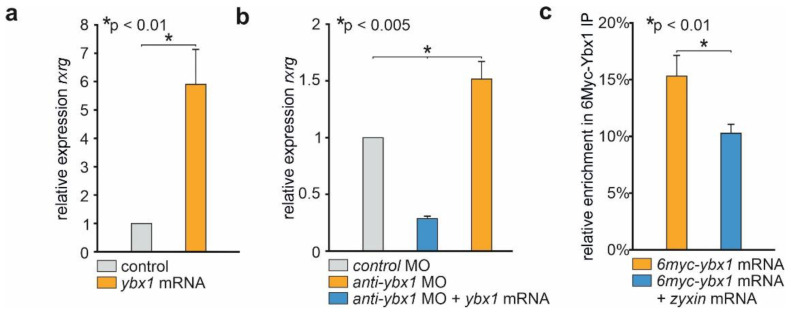
Zyxin regulates the stability of *rxrγ* mRNA by preventing formation of the Ybx1/mRNA complex. (**a**) Overexpression of *ybx1* leads to an increase in the *rxrγ* mRNA level. (**b**) Suppression of *ybx1* mRNA translation by *anti-ybx1* MO decreases the *rxrγ* mRNA level, while co-expression of *ybx1* mRNA restores it, even with some overshooting. (**c**) RIP experiments demonstrate the ability of Ybx1 to bind *rxrγ* mRNA, whereas Zyxin reduces this ability. Error bars indicate standard deviation. *p*-value was measured by Student’s *t*-test. The data presented are averaged from at least three independent experiments.

To test if Ybx1 could regulate the *rxrγ* mRNA level, we overexpressed it in embryos by injecting the synthetic *ybx1* mRNA. Indeed, a strong increase in *rxrγ* transcripts concentration was revealed by qRT-PCR in these embryos comparing to the control ones (Figure 2a). The opposite effect was obtained when *anti-ybx1* MO was injected (Figure 2b). 

Next, we tested if Ybx1 is able to bind to *rxrγ* mRNA to protect it from degradation, using RNA-immunoprecipitation assay (RIP). *Xenopus* embryos were injected with myc-tagged *ybx1* mRNA and *zyxin* mRNA, and lysed at stage 11. The lysates were incubated with anti-myc resin and the precipitated material was analyzed by qPCR as described in [22]. In the samples with expressed myc-Ybx1 we observed strong enrichment of *rxrγ* mRNA comparing to a control sample, injected with myc-tagged mRNA of a secreted morphogen Chordin. On the other hand, in the sample with co-expressed both myc-*ybx1* and *zyxin* mRNA we observed a decrease in the amount of *rxrγ* mRNA. Therefore, we conclude that Ybx1 does form a complex with *rxrγ* mRNA, and that Zyxin can counteract to the complex formation (Figure 2c). 

### 2.3. Downregulation of Zyxin Results in the Activation of Luciferase Reporters Containing the Cis-Regulatory Elements Essential for the RA-Dependent Signaling

**Figure 3:**
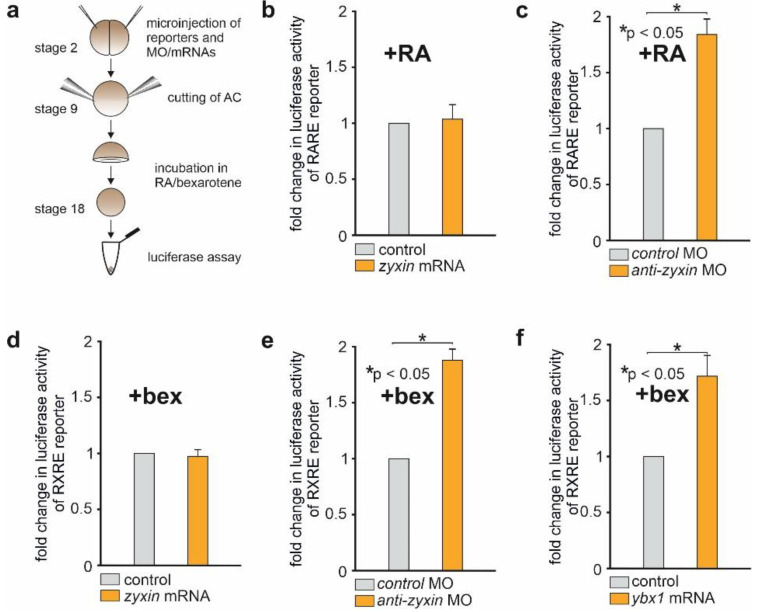
Effects of loss- and gain-of-function of *Zyxin* on γRXRE and RARE cis-regulatory elements in the luciferase reporter assay. (**a**) Experimental design. (**b**) *Zyxin* overexpression does not result in the activation of *pGL3-3xRARE* reporter in the presence of RA. (**c**) *pGL3-3xRARE* reporter activity induced by RA increased in AC with the downregulated *zyxin*. (**d**) *Zyxin* overexpression does not result in the activation of *pGL3-2xγRXRE* reporter in the presence of bexarotene. (**e**) *pGL3-2xγRXRE* reporter activity induced by bexarotene increased in AC with the downregulated *Zyxin*. (**f**) *pGL3-2xγRXRE* reporter activity increased in AC with the overexpressed *ybx1* in the presence of bexarotene. Error bars indicate standard deviation. *p*-value was measured by Student’s t-test. The data presented are averaged from at least three independent experiments.

In order to determine the Zyxin influence on the RA-dependent signaling cascade, we designed a luciferase reporter containing RA-responsive cis-regulatory elements (RARE) upstream of the luciferase gene. To do so, we cloned the 3xRARE sequence (3xGGGTAGGGTTCACCGAAAGTTCACTCG) [23] into the pGL3-Promoter Vector (Promega) plasmid containing the SV40 promoter upstream of the luciferase sequence. We also created similar constructs with 6xRARE and 12xRARE elements in the same plasmid. The efficiency of these constructs was tested as follows: a mixture of the specific reporter *pGL3-Promoter Vector-3xRARE/6xRARE/12xRARE* and the Renilla reporter under the control of a low-specific thymidine kinase promoter was injected into the animal region of each blastomere of *Xenopus laevis* embryos at the 2–4 blastomere stage. Animal caps (AC) were excised at the stage of late blastula (stage 9) and harvested at stage 18. ACs were incubated in 1x Modified Marc’s Ringer’s (MMR) with 10^−5^ M retinoic acid. Control ACs were incubated in 1xMMR only (Figure 3a). Interestingly, samples injected with *pGL3-Promoter Vector-3xRARE* demonstrated the strongest luciferase activity in response to retinoic acid. Multiplication of the RARE elements did not increase activity, but somewhat weakened it (Figure S3a). In the following experiments we used the 3xRARE construct.

To investigate the effect of Zyxin on this signaling, we co-injected *pGL3-3xRARE* and *Renilla* reference reporter with *anti-zyxin* MO, *zyxin* mRNA or *control* MO into the embryos, treated their AC with 10^−5^ M RA and then measured the luciferase signal (Figure 3a). As a result, we observed an increased expression of *pGL3-3xRARE* in AC co-injected with *anti-zyxin* MO, but not with *zyxin* mRNA (Figure 3b–d). 

Although the data obtained by means of *pGL3-3xRARE* reporter were in agreement with the data indicating the role of Zyxin in the *rxrγ* mRNA destabilization, there was still some uncertainty in the direct involvement of Rxrγ in the RA signaling regulation by Zyxin, since the retinoic receptor heterodimer, which must be formed for RARE activation, includes besides Rxr its Rar counterpart [13]. To resolve this uncertainty, we created a reporter with the luciferase gene driven by γRXRE regulatory elements sensitive exclusively to the RXR homodimer [24]. To this end, we cloned 2xγRXRE sequences (2xGGTTGAaAGGTCA) into the *pGL3-Promoter Vector*. The effectiveness of the resulting construct (*pGL3-2xγRXRE*) was verified in the AC explants in the same manner as the effectiveness of *pGL3-3xRARE* reporter, except 10^−5^ M solution of the Rxrs agonist bexarotene was used for the activation of *pGL3-2xγRXRE* (Figure S3b). Accordingly, bexarotene was also used in all other experiments with this reporter. 

As a result, we established that similarly to the *pGL3-3xRARE*, injections of *anti-zyxin* MO or synthetic *ybx1* mRNA significantly increased *pGL3-2xγRXRE* expression, whereas *Zyxin* overexpression did not affect the activity of this reporter (Figure 3d–f).

In addition, the activity of reporters was only changed when retinoic acid (for RARE) or bexarotene (for RXRE) was added to the medium (compare Figure 3c with Figure S4b, and Figure 3e with Figure S4d). In the absence of an agonist, the receptor itself is unable to form dimers and bind to regulatory elements. It is evidently apparent from experiments with *rxrγ* overexpression, since only when bexarotene was added did the activity of the reporter increase severalfold; without the agonist, it increased slightly (Figure S3b).

In sum, all these results confirm the functional role of the previously found mechanism of Zyxin influence on the degradation of maternal *rxrγ* mRNA.

### 2.4. The Distribution of Zyxin between the Cytoplasm and the Nucleus Is Not Changed upon Treatment of AC with RA or Bexarotene

Recently, Youn et al., [25] have shown that in cell culture Zyxin can shuttle into the nucleus from cytoplasm upon stimulation with RA. In this regard, we decided to verify whether Zyxin also moves into the nucleus upon RA or the Rxrγ receptor agonist, bexarotene, stimulation in the animal cap (AC) explants of *Xenopus* embryos. To this end, we incubated AC explants in 10^−5^ M RA or bexarotene, lysed these explants to separate nuclei from cytoplasm, and analyzed the two fractions by Western blotting. As a result, we revealed that neither RA nor bexarotene affect Zyxin distribution between the cytoplasm and nucleus (Figure S5). 

## 3. Discussion

In this study, we demonstrate that *Zyxin* down-regulation up-regulates the level of *rarγ* mRNA, but not *rarγ* mRNA, in the *Xenopus laevis* embryo. Zyxin-dependent regulation of *rxrγ* mRNA relies on the previously reported mechanism of mRNA stabilization by association with RNA-binding protein Y-box factor 1 (Ybx1) [10]. Zyxin can bind to Ybx1 and sequester it from the target *rxrγ* mRNA, resulting in its accelerated degradation. The following line of evidence for these conclusions was obtained. First, we demonstrated that Ybx1 can precipitate *rxrγ* mRNA, whereas addition of Zyxin significantly decreases the co-precipitation of *rxrγ* transcripts. Secondly, Ybx1 overexpression significantly increases the endogenous *rxrγ* mRNA level, while downregulation of Ybx1 has the opposite effect. Thirdly, the knock-down of *Zyxin* by MO, as well as Ybx1 overexpression, activates RARE and γRXRE-containing luciferase reporters. However, overexpression of *Zyxin* does not activate them. The latter is consistent with our previous data, showing that *Zyxin* overexpression has a minor effect on the developing embryo [7,10]. Finally, we demonstrated that the effect of *rxrγ* mRNA increase upon *Zyxin* down-regulation is also reproduced in the *Danio rerio* fish embryos, which indicates a conservation of the revealed mechanism of the regulation of *rxrγ* mRNA stability by Zyxin among vertebrates. 

Evidence of the functional relationship between retinoic acid receptors (Rars), retinoid X receptors (Rxrs), and the cytoskeletal LIM domain proteins of Zyxin family has periodically appeared in the literature over the past decade [26]. The Zyxin family proteins Ajuba, Limd1, and WTIP have been shown to directly interact with RAR and RXR receptors as corepressors of RARE-dependent transcription. Generally, retinoid nuclear receptor corepressors bind directly to that receptor and are thought to act as scaffolds to recruit chromatin remodeling complexes to specific targets [27]. In the same work it was shown that Zyxin and LPP do not interact with RAR and RXR receptors and do not suppress the activity of RARE-driven promoters. In the present work, we also confirmed that *Zyxin* overexpression does not affect RARE and RXRE-dependent transcription. 

At the same time, to our knowledge, there was the only evidence of interaction of Zyxin and RA signaling until hitherto. Namely, in H1299 cell culture, Zyxin moves into the nucleus in the presence of RA and represses RARE-dependent transcription forming a triple complex with PTOV1 and CBP, a co-activator of RARE, by sequestering CBP from RARE [25].

Obviously, in our present work, we have identified a completely different mechanism of the regulation of RA signaling by Zyxin based on the destabilization of *rxrγ* mRNA. In addition to all the functional data, this is also confirmed by the fact that, in contrast to the work of Youn et al., [25], we did not detect the movement of Zyxin into the cell nuclei in response to treatment of animal caps with RA or bexarotene. Taking into account that we recently showed the involvement of the same mechanism in the regulation of the expression of embryonic stemness genes [10], one may assume that it can generally take part in the control of the expression of a fairly wide range of maternal genes. In this regard, a large-scale search for genes whose expression is influenced by this mechanism would be an interesting prospect in the future.

## 4. Materials and Methods

The materials used in the work are outlined in Table 1.

### 4.1. Experimental Model and Subject Details

#### Embryo Manipulations

The care and handling of all animals was conducted in accordance with the regulations of the Animal Committee at the Shemyakin-Ovchinnikov Institute of Bioorganic Chemistry (Moscow, Russia), and the Animals (Scientific Procedures) Act of 1986, and the Helsinki Declaration. 

*Xenopus laevis* embryos were obtained with HCG injection as described by [28]. The embryos were staged as described by Nieuwkoop and Faber [29].

The D. rerio (AB/TL strain) was raised in E3 medium (5 mM NaCl, 0.17 mM KCl, 0.33 mM CaCl_2_, 0.33 mM MgSO_4_, adjusted to pH 7.0) at 26.5 °C with 12 h/12 h of light and dark.

### 4.2. Method Details

#### 4.2.1. Embryonic Microinjections

Microinjections were performed as described previously [10,22,28]. 

#### 4.2.2. mRNA Extraction and qRT-PCR

*Xenopus laevis* embryos were extracted using ExtractRNA reagent (Evrogen) and CleanRNA Standard spin columns (Evrogen) following manufacturer’s instructions.

Nearly 250 ng of total RNA were transcribed into cDNA using MMLV RT kit (Evrogen), and PCR was performed with qPCRmix-HS SYBR kit (Evrogen). Amplifications were conducted using DTprime 4 qPCR amplifier (DNA-Technology). We also amplified the housekeeping genes *odc1* and *eef1a* to evaluate RNA quality. See Table S1 for primers we used.

We used stage 11 to study the amount of endogenous maternal mRNAs, since after the activation of the embryonic genome at the blastula-early gastrula stage, maternal transcripts rapidly degrade, and the greatest difference in the number of transcripts is observed precisely at stage 11.

#### 4.2.3. DNA Constructs

Previous studies have described the following plasmids: *p35T-Zyxin* and *pCS2MT-Ybx1* [7,10]. 

In Table S1, we provide detailed cloning strategies for *pGl3pv -RARE*, *pGl3pv -RXRE*, and *pCS2-rxrγ-3xmyc*.

#### 4.2.4. Synthetic mRNA

After linearizing pCS2-based plasmids with NotI, synthetic mRNAs (see Table S1) were prepared with the mMessage mMachine SP6 kit (Ambion) and injected into 2–4 cell embryo (3–4 nL of 100ng/mL mRNA water solution per embryo) into the whole embryo or into a particular blastomere depending on the experimental design.

#### 4.2.5. RNA-Immunoprecipitation (RIP)

To study the ability of Ybx1 to bind *rxrγ* mRNA, 2-cell stage embryos were injected with *myc-ybx1* RNA (35 pg/blastomere) and *zyxin* RNA (50 pg/blastomere). RIP analysis was performed according to RIP protocol (Martynova et al., 2021b).

#### 4.2.6. Luciferase Reporter Assay

The Luciferase assay was performed following the instructions [30]. To check reporter activity, *pGl3pv–RARE* or *pGl3pv–RXRE* plasmid (50 pg/blastomere) and reference *pRL-TK-renilla* luciferase plasmid (Promega) (50 pg/blastomere) mixed with 0.3 mM *anti-zyxin* MO or *ybx1* mRNA (35 pg/blastomere) were injected to each blastomere of 2-cell stage embryos. Incubation of the injected embryos in 0.1 × MMR was continued until the late blastula stage (stage 9). Then explants of animal caps (ACs) were cut out with two tweezers. The ACs were then placed in 1 × MMR for two hours. Then, depending on the experiment, 10 µM RA or bexarotene was added to the medium.

The AC were collected for luciferase activity detection at stage 18. The problem is that when using whole embryos, the yolk gets into the lysate, strongly dampening the luciferase signal unspecifically. For the purity of the experiment, we have to carry out all studies on luciferase reporters on animal caps. It takes some time to accumulate a sufficient amount of reporter protein (luciferase) since reporter constructs (plasmids) can begin to be transcribed only after the embryonic genome (MBT) has been activated. Stage 11 (middle gastrula) contains very little reporter protein, which makes it difficult to interpret the findings. Therefore, all luciferase tests are carried out on animal caps incubated until stages 16–18 of development.

#### 4.2.7. Cytoplasmic and Nuclear Fractionation

For our purposes, Xenopus embryos were incubated in 0.1 × MMR with 10 μM RA/bexarotene from stage 9 to stage 18. AC were cut out at stage 9 and incubated in 1 × MMR with 10 μM RA/bexarotene till stage 18.

Embryos or AC were pretreated by incubation in 0.1 × MMR/1 × MMR containing 150 μg of cycloheximide per ml. After incubation for 1 h at 23 °C, nuclear and cytoplasmic fractions were prepared as described in [28].

#### 4.2.8. Statistical Analysis

For single comparisons standard t-test was used. For multiple comparisons ANOVA test was used. The statistical analysis was performed in GraphPad Prism.

## Figures and Tables

**Table 1:** Key resources table.

Reagent or Resource	Source	Identifier
Antibodies		
Rabbit polyclonal anti-Zyxin	A.G. Zaraisky lab, Moscow, Russia	N/A
Anti-rabbit IgG, AP-conjugated, produced in goat	Sigma-Aldrich, Missouri, USA	Cat#A3937; RRID:AB_258122
Mouse monoclonal anti-alpha-Tubulin	Sigma-Aldrich, Missouri, USA	Cat#T9026; RRID:AB_477593
Anti-mouse IgG, AP-conjugated	Sigma-Aldrich, Missouri, USA	Cat#A3562; RRID:AB_258091
EZview Red Anti-c-Myc Affinity Gel	Sigma-Aldrich, Missouri, USA	Cat#E6654; RRID:AB_10093201
Bacterial and Virus Strains		
DH5alpha *E. coli* strain	N/A	N/A
Chemicals, Peptides, and Recombinant Proteins		
Human chorionic gonadotropin	Sigma-Aldrich, Missouri, USA	Cat#CG10
Ethyl 3-aminobenzoate methanesulfonate salt	Sigma-Aldrich, Missouri, USA	Cat#A5040
Western Blue^®^ stabilized substrate for alkaline phosphatase	Promega, Madison, Wisconsin, USA	Cat#S3841
BM Purple AP-substrate	Roche, Basel, Switzerland	Cat#11442074001
Brilliant Blue G solution	Sigma-Aldrich, Missouri, USA	Cat#B8522
Cycloheximide	Sigma-Aldrich, Missouri, USA	Cat#C1988
CNBr-Activated Sepharose^TM^ 4B	GE Healthcare, Chicago, Illinois, USA	N/A
Fluorescein Lysine Dextran (FLD) 40 kD	Invitrogen, Waltham, Massachusetts, USA	Cat#D1845
L-Cysteine	Dia-m, Moscow, Russia	Cat#M52904
Ficoll	Dia-m, Moscow, Russia	Cat#PS400
Igepal Nonidet P-40	Sigma-Aldrich, Missouri, USA	Cat#I-30201
DTT	Fermentas, Waltham, Massachusetts, USA	Cat#RO861
RNase Out RNase inhibitor, 100 units/ml	Invitrogen, Waltham, Massachusetts, USA	Cat#10777-019
Vanadyl ribonucleoside complexes (VRC)	New England Biolabs, Ipswich, Massachusetts, USA	Cat#S1402S
Protease Inhibitor Coctail	Sigma-Aldrich, Missouri, USA	Cat#P8340
Bexarotene	LLC “Gurus BioPharm”, Moscow, Russia	N/A
Retinoic acid (RA)	Sigma-Aldrich, Missouri, USA	Cat#R2625
Critical Commercial Assays		
mMessage mMachine^TM^ SP6kit	Thermo Fisher, Waltham, Massachusetts, USA	Cat#AM1340
ExtractRNA	Evrogen, Moscow, Russia	Cat# BC032
CleanRNA Standard	Evrogen, Moscow, Russia	Cat# BC033
NucleoSpin RNA purification columns	Macherey Nagel, Germany	Cat#REF 740955.50
MMLV RT	Evrogen, Moscow, Russia	Cat# SK021
qPCRmix-HS SYBR	Evrogen, Moscow, Russia	Cat# PK147L
Dual-Luciferase Reporter Assay System	Promega, Madison, Wisconsin, USA	Cat# E1910
Dynabeads mRNA DIRECT Kit	Ambion, Waltham, Massachusetts, USA	Cat# 61011
Experimental Models: Organisms/Strains		
Wild type *Xenopus laevis* frogs	Nasco, Chicago, USA	Cat#LM00456; RRID:XEP_Xla100
Danio rerio AB/TL strain	N/A	N/A
Oligonucleotides		
See Table S1 for all oligonucleotides used		
Recombinant DNA		
See Table S1 for all recombinant DNAs used		
Software and Algorithms		
Excel	Microsoft, USA	N/A
ImageJ	Schneider et al., 2012	https://imagej.nih.gov/ij/
Other		
See Table S1 for all recombinant DNAs and oligonucleotides used		

## Supplementary Materials

The following supporting information can be downloaded at: https://www.mdpi.com/article/10.3390/ijms23105627/s1.
