# Supplementary material for: The Cytoskeletal Protein Zyxin Inhibits Retinoic Acid Signaling by Destabilizing the Maternal mRNA of the RXRγ Nuclear Receptor
Source: Int J Mol Sci. 2022 May 17;23(10):5627. doi: 10.3390/ijms23105627 (PMC9147113; doi:10.3390/ijms23105627)
Supplement: Supplementary file 1 [file ijms-23-05627-s001.zip › ijms-1711914-supplementary.pdf]

**SUPPLEMENTAL INFORMATION**

**Supplementary Table**

**Table S1: morpholino sequences, qRT-PCR primers and plasmids cloning strategies. Related to STAR Methods**

| **Name** | **Description and sequences of the morpholino oligonucleotides** |
|---|---|
| *zyxin X* MO1 | A common MO to positions +32–+57 of Zyxin mRNA of both Xenopus laevis zyxin pseudoalleles:<br>TGAAATGTTGATGGTGAAGGAGGAC |
| *zyxin X MO2* | A common MO to positions 0–+25 of Zyxin mRNA of both Xenopus laevis Zyxin pseudoalleles:<br>GGGTGGCAGGAGCCGCTGGGTCCAT |
| *zyxin splice X MO* | A common MO to positions +953–+972 of Zyxin mRNA of both Xenopus laevis Zyxin pseudoalleles:<br>ctcccactactcacaactcaCCTGATG<br>(intron) (exon) |
| control MO | A mismatched variant of *zyxin* MO<br>TTAACTGTTTAATGTTGAATGAGAAC<br>as a negative controls. |
| *zyxin Danio MO1* | MO to positions -23–+3 of Danio Zyxin mRNA :<br>CATCTTGATTGTTCGTTTTCTTCGT |
| | |
| **qRT-PCR primers:** | |
| **Name** | **Description and sequences of the oligonucleotides** |
| ***Xenopus laevis*** | |
| *ornithine decarboxylase 1 (odc1)* | Forward primer: 5'-GCCAGTTCTAACAAAGAAACCCA<br>Reverse primer: 5'-TCTACGATACGATCCAGCCCA<br>Product length: 93 |
| *eukaryotic translation elongation factor 1 alpha 1 (eef1a1)* | Forward primer: 5'-GTTCATTTACCGCACAGGTTATCA<br>Reverse primer: 5'-ACACAGGGGCATATCCAGCA<br>Product length: 70 |
| *retinoic acid receptor gamma (rarg)* | Forward primer: 5'-GTCGACGAAGGCCTGACAAA<br>Reverse primer: 5'-GAATCAGCGGAGGCATAGGT<br>Product length: 141 |
| *retinoid X receptor gamma (rxrg)* | Forward primer: 5'-CTTCCTTCTCGCACCGCTC<br>Reverse primer: 5'-GCCTACTCCCGCATTGTGT<br>Product length: 98 |

| *Danio rerio* | |
|---|---|
| *ornithine decarboxylase 1 (odc1)*<br><br><br><br>*elongation factor 1-alpha (ef1a)*<br><br><br><br><br><br>*retinoid X receptor gamma (rxrg)* | Forward primer: 5'-CTCCACCTTCAATGGCTTCCAG<br>Reverse primer: 5'-AGTGGGATGGCACGTTTCCAG<br>Product length: 87<br><br>Forward primer: 5'-AAGAACGTGTCAGTCAAGGACAT<br>Reverse primer: 5'-CGTAACCCTGAGAGATCTGACCA<br>Product length: 76<br><br>Forward primer: 5'-AGCAATCGTCCTCTTCAACCC<br>Reverse primer: 5'-TTGTGTTTGGTGTAGCCCTCC<br>Product length: 108 |
| **DNA constructs** | **PCR primers and cloning strategy used to prepare reporter plasmids.** |
| *pGl3pv -3xRARE* | 1.The insert was obtained by annealing two primers:<br>with forward primer 5'-CGGGTAGGGTTCACCGAAAGTTCACTCGGGGTAGGGTTCACCGAAAGTTCACTCGA<br>and reverse primer<br>5'-GATCTCGAGTGAACTTTCGGTGAACCCTACCCCGAGTGAACTTTCGGTGAACCCTACCCGGTAC<br>2.Cloning by KpnI and BglII into KpnI and BglII of *pGl3 promoter* vector (Promega); checking by sequencing. |
| *pGl3pv -6xRARE, pGl3pv -12xRARE* | 1.PCR from *pGl3pv -3xRARE*<br>with forward primer 5'- aatt gtcgacCTAGCAAAATAGGCTGTCCC<br>and reverse primer 5'- aatt ctcgagCTAAATGAGATGCAGATCGC<br>2.Cloning by SalI and XhoI into XhoI of *pGl3 promoter vector* (Promega), preprocessed by CIAP.<br>3. Checking by sequencing. |
| *pGl3pv - RXRE* | 1. The insert was obtained by annealing two primers:<br>with forward primer 5'-gtaccTGGGGTTGAAAGGTCAGATGGATGGGGTTGAAAGGTCAGATGGAa<br>and reverse primer<br>5'-gatctTCCATCTGACCTTTCAACCCCATCCATCTGACCTTTCAACCCCAg<br>2.Cloning by Acc65I and BglII into Acc65I and BglII of *pGl3 promoter* vector (Promega); checking by sequencing. |

| **Synthetic mRNA** | **PCR primers and cloning strategy used to prepare DNA templates for generation of synthetic mRNA.** |
|---|---|
| *rxrg-3xmyc* | 1.PCR from the total *X. laevis* cDNA stage 8<br>with forward primer 5’- ATGCATCTTGCTACTGAGACA<br>and reverse primer 5’- TCATGAGATCTGGTGAGGTG<br>2.The obtained cDNA fragment was cloned into pAL2-T Vector (Evrogen) and verified by sequencing. Construct: pAL2T-rxrg<br>3. PCR from pAL2T-rxrg<br>with forward primer 5’- aatt aggcctGCCGCCACCATGCATCTTGCTACTGAGACA<br>and reverse primer 5’- aatt ctcgagTGAGATCTGGTGAGGTGTTT<br>4.Cloning by Eco147I and XhoI into EcoRI-Klenow and XhoI of pCS2-3xmyc; checking by sequencing. Final construct: pCS2-rxrg-3xmyc. |
| *Zyxin;*<br>*Myc-Ybx-1 ;* | *p35T-Zyxin; pCS2MT-Ybx1* plasmids were obtained as described (Martynova et al, 2008; Parshina et al., 2020) |

## Supplementary Figures

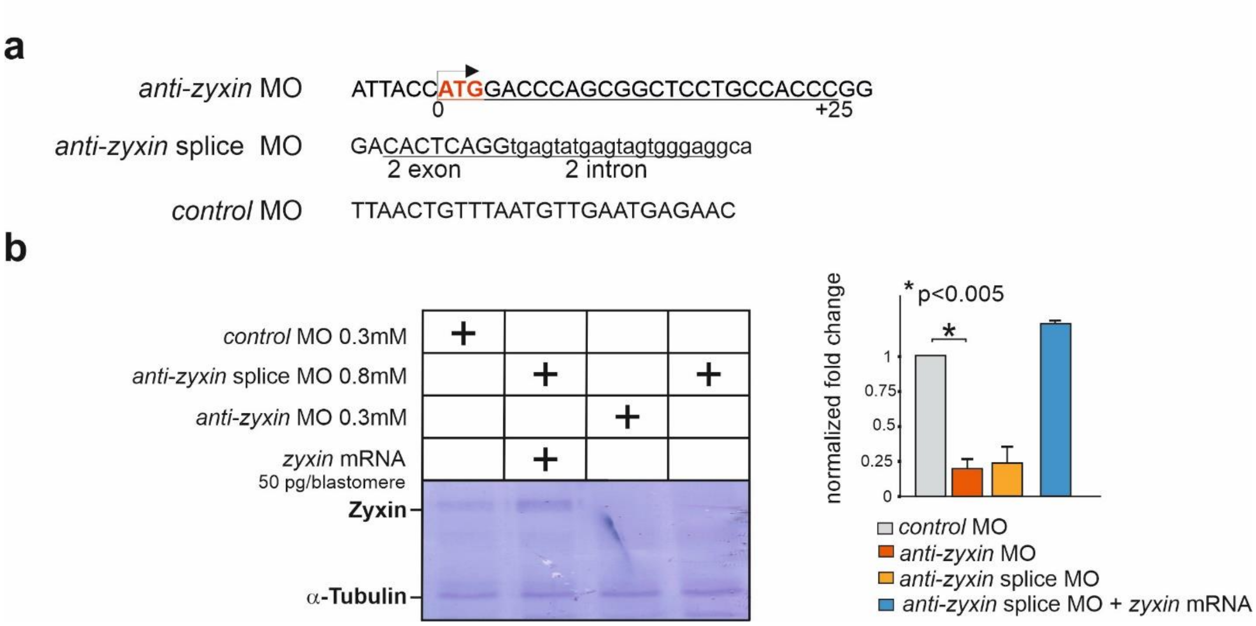


**Figure S1. Testing of MO specificity and efficiency.**

**(a)** Scheme of the morpholino target sites on X. laevis zyxin mRNA.

**(b)** For testing the specificity, MOs were injected into each blastomere of 2-cell X. laevis embryos in different concentrations: 0,3mM (for anti-zyxin and control MO); 0,8mM for anti-zyxin splice MO and 0,8mM for anti-zyxin splice MO with 50 pg/blastomere zyxin mRNA.

The injected embryos were collected at the middle gastrula stage and analyzed for amount of Zyxin by Western blotting with anti-Zyxin antibody (Martynova et al., 2008). The data presented are representative of at least three independent experiments.

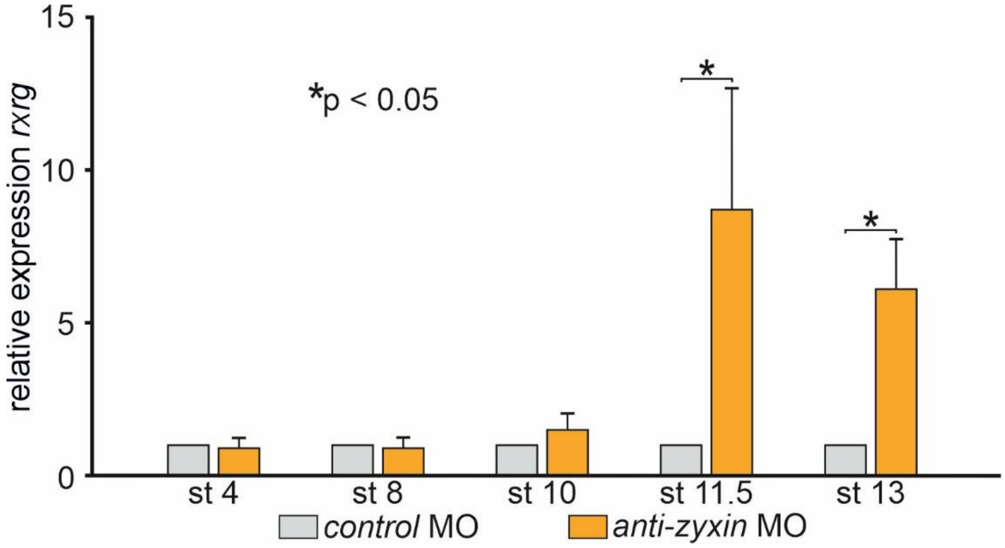


**Figure S2. Analysis by qRT-PCR of *rxrγ* expression.**

Comparative analysis of the *rxrγ* transcripts levels in *zyxin* knocked-down and control embryos at different stages of development. This data corresponds to the data presented in Figure 1d. Error bars indicate standard deviation. P-value was measured by Student’s t-test.

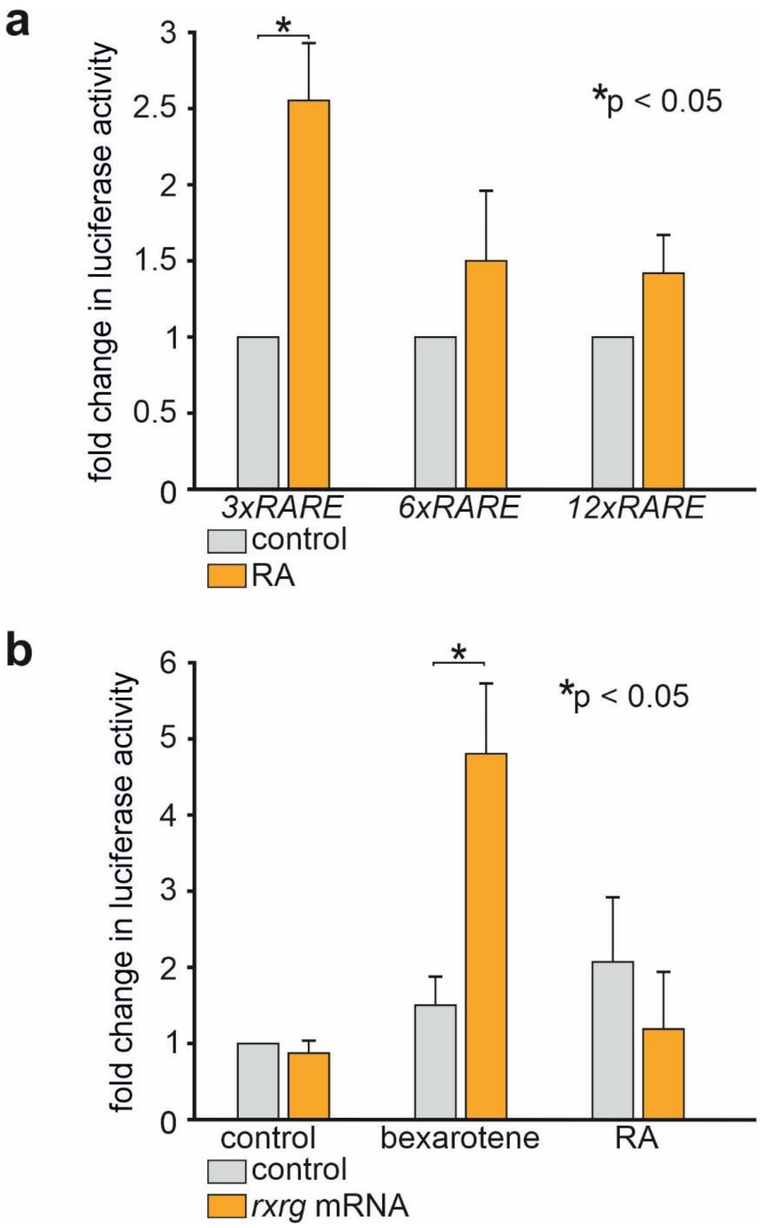


**Figure S3. Testing of RARE and RXRE reporters on the AC explants**

- **(a)** Activation of *pGL3-Promoter Vector-3xRARE*/*6xRARE/12xRARE* in response to retinoic acid.
- **(b)** *pGL3-Promoter Vector-gRXRE* reporter activity was compared between experimental samples with *rxrg* overexpression and control samples. ACs were incubated in solutions with the RXRs agonist bexarotene or all-trans RA.
  The data presented are representative of at least three independent experiments.

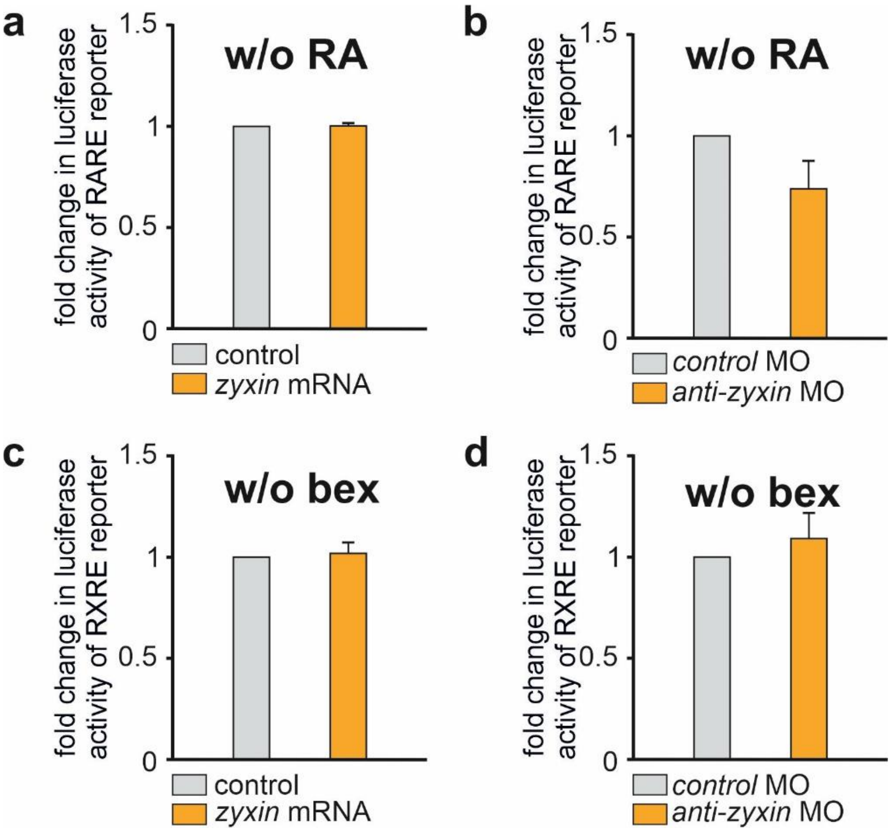


**Figure S4. Effects of loss- and gain-of-function of zyxin on γRXRE and RARE cis-regulatory elements in the luciferase reporter assay in the absence of RA or bexarotene.**

- **(a)** Zyxin overexpression does not result in the activation of *pGL3-3xRARE* reporter in the absence of RA.
- **(b)** Zyxin knockdown does not result in the activation of *pGL3-3xRARE* reporter in the absence of RA.
- **(c)** Zyxin overexpression does not result in the activation of *pGL3-2xγRXRE* reporter in the absence of RA.
- **(d)** Zyxin knockdown does not result in the activation of *pGL3-2xγRXRE* reporter in the absence of RA.

The data presented are averaged from at least three independent experiments.

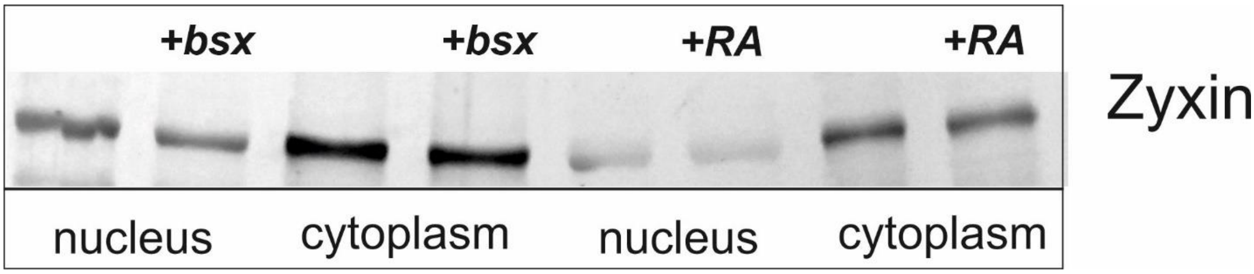


**Figure S5. RA and bexarotene do not affect the distribution of Zyxin between the nucleus and the cytoplasm**

AC explants were incubated in 1x MMR with $10^{-5}$M bexarotene (lanes 1-4) or $10^{-5}$M RA (lanes 5-8). Explants were lysed at stage 18 to separate the nuclei from the cytoplasm, and the resulting fractions were analyzed by Western blotting. The data presented are representative of at least three independent experiments.
